# Participation of Latinos in the Diabetes Self-Management Program and *Programa de Manejo Personal de la Diabetes*

**DOI:** 10.1093/geroni/igaa006

**Published:** 2020-03-18

**Authors:** Carolyn A Mendez-Luck, Diana J Govier, Jeff Luck, Esmeralda J Julyan, Shyama Mahakalanda, Angelica P Herrera-Venson

**Affiliations:** 1 Health Policy and Management, Oregon State University, Corvallis; 2 Center for Healthy Aging, National Council on Aging, Washington, District of Columbia

**Keywords:** Chronic disease, Cultural adaptation, Disparities, Ethnicity, Linguistic adaptation

## Abstract

**Background and Objectives:**

The Diabetes Self-Management Program (DSMP) and *Programa de Manejo Personal de la Diabetes* (PMPD) have been shown to reduce complications from poorly controlled diabetes. Only a few research studies have examined Latino individuals’ participation in them. This study examines workshop completion among DSMP and PMPD participants and the effects of race/ethnicity, workshop language, workshop type, and workshop site on program completion rates by participants.

**Research Design and Methods:**

We used data from the National Council on Aging’s data repository of individuals who participated in DSMP or PMPD between January 2010 and March 2019. Using a pooled cross-sectional study design, we examined workshop completion among 8,321 Latino and 23,537 non-Latino white (NLW) participants. We utilized linear probability models to estimate the effects of race/ethnicity and workshop language/type among the full sample; a stratified model estimated the separate effects of workshop language, type, and delivery site among Latinos. Participant characteristics included age, sex, education, number of chronic health conditions, living arrangement, health insurance status, and geographic location of workshop.

**Results:**

Compared to NLW participants in DSMP English workshops, Latinos enrolled in any workshop had a higher probability of completing at least four workshop sessions, and Latinos enrolled in PMPD Spanish workshops had a higher probability of completing six of six sessions. Among the Latino subsample, participation in PMPD Spanish or English workshops was associated with completing at least four sessions or all six sessions compared with participation in DSMP Spanish or English workshops. Among Latino participants, the effects of workshop site on completion rates were mixed.

**Discussion and Implications:**

Diabetes self-management education programs tailored for Latino participants had higher completion rates. Further research is warranted to better understand the effect of workshop site and participant characteristics on completion of DSMP and PMPD programs.

**Translational Significance:** A Spanish-language Diabetes Self-Management Program was designed for community-dwelling Latino adults with type 2 diabetes to improve Latino adults’ health status and self-management skills. The program was subsequently developed in English and offered to non-Latinos. Among white participants of both programs, those who identified as Latino were more likely to complete 60–100% of sessions than non-Latino participants. Spanish language delivery was associated with higher completion rates by Latino participants, suggesting that linguistically appropriate curricula increase participants’ interest in program content.

## Background and Objectives

The prevalence of type 2 diabetes in the U.S. Latino population is twice that observed among non-Latino Whites (NLW) (Centers for Disease Control [CDC], 2017), and over half of U.S. Latinos are expected to develop type 2 diabetes by age 70 (Schneiderman et al., 2014). Poorly controlled diabetes imposes excess morbidity and financial hardship on Latinos. Latino adults also have higher incidence of complications resulting from uncontrolled diabetes, such as nephropathy, retinopathy, and cardiovascular disease, than do NLW or African American adults (Lanting, Joung, Mackenbach, Lamberts, & Bootsma, 2005; Osborn, de Groot, & Wagner, 2013). Latinos with diabetes incurred an average of $8,050 in 2017 for health care expenditures associated with having diabetes (American Diabetes Association, 2018).

Diabetes self-management education can effectively reduce complications from poorly controlled diabetes (CDC, 2018). Diabetes self-management education is designed to facilitate knowledge and diabetes self-care, and has been linked to reduced medical costs and all-cause health care use (Turner, Ma, Lorig, Greenberg, & DeVries, 2018). Typically, such programs focus on reinforcing and sustaining behaviors needed to manage diabetes, specifically glycemic control (Beck et al., 2017; Mayberry & Osborn, 2012; Wen, Parchman, & Shepherd, 2004; Wen, Shepherd, & Parchman, 2004). Some diabetes self-management programs are personalized to the needs of the individual through evidence-based standards by a diabetes educator (CDC, 2018).

Diabetes self-management education has been shown to improve healthy eating, hyperglycemia symptoms, depression, and communication with physicians (Lorig, Ritter, Villa, & Armas, 2009; Ory et al., 2013). The benefits of diabetes self-management education have been observed across multiple measures, including 6 months and 1 year after the intervention (Lorig et al., 2016; Ory et al., 2013). For example, Ory and colleagues (2013) examined data from the National Study of the Chronic Disease Self-Management Program and found that study participants reported more physical activity and fewer emergency room visits and hospitalizations from baseline to 6 months postintervention.

The *Programa de Manejo Personal de la Diabetes* (PMPD; originally called the Spanish Diabetes Self-Management Program) was intentionally designed in Spanish for community-dwelling, Spanish-speaking Latino adults with type 2 diabetes (Lorig, Ritter, Villa, & Piette, 2008; Self-Management Resource Center [SMRC], n. d.). The PMPD is aimed at improving Latino adults’ health status and self-management skills. The Diabetes Self-Management Program (DSMP) was subsequently developed in English from the original PMPD (SMRC, n.d.). The PMPD and DSMP consist of small group workshops of 12–16 participants who take part in 2.5-hr weekly sessions for six consecutive weeks. Workshops cover a wide range of topics, including techniques for coping with diabetes symptoms and managing diabetes medications, and approaches to healthy eating. Program curricula are reviewed and updated annually to meet the standards of the American Diabetes Association (CDC, 2018). Both programs have been shown to be effective in helping individuals with diabetes. An evaluation of PMPD, as part of a randomized community-based outcome trial, showed improved health status, health behavior, self-efficacy, and fewer visits to the emergency room at 4 months and at 1 year when compared with usual-care control participants (Lorig, Ritter, & Gonzalez, 2003). A randomized control trial of DSMP had similar results (Lorig et al., 2009).

Despite widespread use of these two evidenced-based programs (Smith et al., 2015), little research has examined Latino individuals’ participation in them. Findings from an analysis of DSMP administrative data (Erdem & Korda, 2014) showed that Latino older adults compared to non-Latino older adults were 33% less likely to complete the program, defined as having attended at least four of six sessions. However, this study did not address the factors associated with DSMP participation or attendance among Latino adults or include a comparison to PMPD. Moreover, the literature is quite limited on Latino participation in diabetes self-management programs in general. A recent study examined geographic and social factors associated with participating in the Chronic Disease Self-Management Program or in DSMP for workshops offered at 144 sites in Illinois (Bobitt, Aguayo, Payne, Jansen, & Schwingel, 2019). It found that shorter travel distances were associated with better attendance among urban participants, and not graduating high school was associated with lower attendance and completion. However, this study was restricted to only one state, included a small number of Latino participants (*n* = 107), and did not examine participation separately for the DSMP program.

Prior research has focused on outcomes associated with self-management or the development and implementation of culturally tailored diabetes interventions for Latino populations (Brown, Garcia, Kouzekanani, & Hanis, 2002; Lorig et al., 2008, 2009), rather than on program participation and completion. Results from one focus group of Latino adults with type 2 diabetes showed that work and family conflicts, the time of day sessions were offered, and transportation were barriers to participating in a community-based diabetes education program (Francis, Noterman, & Litchfield, 2014). Another qualitative study of 15 Latino patients in a community health clinic showed similar barriers: not having the time, available childcare, or transportation kept patients from participating in the clinic’s diabetes self-management program (Testerman & Chase, 2018). Although Latinos with diagnosed diabetes are less likely ever to attend a diabetes self-management class compared to their same-age white or black counterparts (Centers for Disease Control, n.d.), research has not examined which characteristics of evidence-based diabetes self-management education programs are associated with Latino adults’ attendance and completion.

We therefore analyzed participant data provided by the National Council on Aging to examine two widely implemented diabetes management education programs, DSMP and PMPD. This article describes completion rates for each program for Latino and NLW participants; and examines the effects of workshop type, workshop language, and delivery site on completion rates.

## Method

This study used a pooled cross-sectional study design to analyze 9 years of participant data from the *Programa de Manejo Personal de la Diabetes* (PMPD) and the Diabetes Self-Management Program (DSMP).

### Data and Sample

The data from this study came from the National Chronic Disease Self-Management Education (CDSME) Database, housed at the National Council on Aging (NCOA). Submission of evidence-based program data is a requirement for grantees funded by the Administration for Community Living through the Prevention and Public Health Fund. This information includes participant demographic and health status data, as well as attendance and program site information. Grantees include a range of state health departments, Area Agencies on Aging, community-based organizations, and health care organizations.

Between January 2010 and March 2019, 7,086 DSMP and 615 PMPD workshops were offered in 45 states and 753 counties. A total of 84,885 individuals participated in a DSMP or PMPD workshop for the first time during this time period. Participants were excluded from our sample if they reported a race category other than white or an ethnicity category other than Latino (*n* = 30,082) or did not report their race or ethnicity (*n* = 9,415). Next, participants were excluded if they attended a workshop in a language other than Spanish or English (*n* = 634) or had missing observations for workshop type (*n* = 7,104). In addition, we excluded participants with missing participant characteristic observations (*n* = 6,609), and those who reported being under 18 or over 110 years of age (*n* = 18). Finally, we excluded NLW participants who reported enrolling in PMPD or a Spanish-language workshop due to small cell size (*n* = 157). Thus, our final analytic sample consisted of 31,858 individuals who enrolled in a DSMP (*n* = 28,247) or PMPD (*n* = 3,611) workshop between January 2010 and March 2019.

### Dependent Variables

Our dependent variables were two measures of workshop completion: (1) attending at least four of six workshop sessions and (2) attending six of six workshop sessions. Attending four or more sessions has been the definition of completion used in prior research and endorsed by the NCOA (Erdem & Korda, 2014). For the first and second definitions of workshop completion, we created binary variables equal to 1 if the participant attended at least four of six or all six workshop sessions, respectively, and 0 otherwise.

### Main Independent Variables

Our analysis focused on four main factors that may affect completion rates: (1) participants’ race/ethnicity (Latino vs NLW), (2) workshop type (PMPD vs DSMP), (3) workshop delivery language (Spanish vs English), and (4) workshop delivery site. Because all NLW participants attended DSMP English workshops, our full model included a categorical variable that described all five observed combinations of workshop type, language of delivery, and race/ethnicity: (1) NLW DSMP English (the reference category); (2) Latino DSMP English; (3) Latino DSMP Spanish; (4) Latino PMPD English; and (5) Latino PMPD Spanish. In the stratified model, a four-category dependent variable (DSMP English, DSMP Spanish, PMPD English, PMPD Spanish) enabled us to examine separately the effects of workshop type and language of delivery among Latino participants only. Workshop site was a categorical variable with eight categories: (1) health care organization; (2) educational institution; (3) faith-based organization; (4) residential facility; (5) senior center; (6) other entity, which included workplace, tribal center, state health department, county health department, municipal government, multipurpose social services organization, and other; (7) other community center, which included library, parks and recreation center, and community center; and (8) unknown.

### Participant Characteristics

Participant characteristics included in all regression analyses were: age, sex, education level, number of chronic health conditions, living arrangement, health insurance status, and geographic location of workshop. Age was a continuous variable indicating the age at which the participant was enrolled in the workshop and ranged from 18 to 110 years. Sex was a binary variable equal to 1 if the participant identified as female, and 0 if they identified as male. Education level was a categorical variable with five response categories: (1) did not graduate high school; (2) high school graduate or GED; (3) some college or technical school; (4) bachelor’s degree or higher; and (5) unknown. Number of chronic conditions was self-reported and ranged from 0 to 15 conditions. Living arrangement was a binary variable equal to 1 if the individual reported living alone, and 0 otherwise. Health insurance status was a binary variable equal to 1 if the participant reported having health insurance, and 0 otherwise. Geographic location was based on the state where the workshop was delivered. We used Census Bureau-designated divisions (U.S. Census Bureau, 2018) to create 10 categories: (1) New England; (2) Mid Atlantic; (3) East North Central; (4) West North Central; (5) South Atlantic; (6) East South Central; (7) West South Central; (8) Mountain; (9) Pacific; and (10) unknown.

### Analytic Approach

We summarized completion rates as well as workshop and participant characteristics of the study sample (Table 1). To test for differences among Latino and NLW participants, we ran chi-squared tests (χ ^2^) for categorical variables and analysis of variance tests (ANOVA) for continuous variables.

**Table 1.** Descriptive Characteristics of Study Sample

	NLW	Latino				
Characteristic	DSMP English	DSMP English	DSMP Spanish	PMPD English	PMPD Spanish	*p* value
*N*	23,537	3,662	1,048	360	3,251	
Percent of sample	73.9%	11.5%	3.3%	1.1%	10.2%	
Percent of Latino sample	—	44.0%	12.6%	4.3%	39.1%	
*Dependent Variables*						
Attended 6 of 6 workshop session	34.5%	19.6%	16.4%	37.5%	32.6%	<.001
Attended 4+ of 6 workshop sessions	75.3%	81.8%	89.7%	83.6%	80.9%	<.001
Mean number of workshop sessions attended	4.4	4.2	4.3	4.7	4.5	<.001
*Independent Variables*						
*Workshop Site*						<.001
Health care organization	22.3%	46.3%	11.9%	17.2%	29.7%	
Educational institution	1.7%	1.2%	1.3%	3.6%	5.3%	
Faith-based organization	5.6%	3.9%	3.2%	8.6%	8.2%	
Residential facility	12.9%	8.7%	9.0%	6.7%	11.7%	
Senior center	24.5%	18.9%	58.6%	38.6%	20.6%	
Other entity	13.0%	8.0%	5.8%	9.7%	11.1%	
Community center	18.2%	12.8%	10.2%	15.6%	13.5%	
Unknown site	1.8%	0.3%	0.0%	0.0%	0.0%	
Mean age	67.8	65.1	69.2	63.5	61.3	<.001
Female	72.7%	73.1%	78.1%	75.3%	76.1%	<.001
Mean number of chronic conditions	3.3	2.2	1.6	2.2	2.2	<.001
Health insurance	86.2%	95.8%	100.0%	94.2%	96.5%	<.001
Lives alone	41.3%	6.9%	75.5%	39.4%	40.9%	<.001
*Education level*						<.001
Some primary school	4.9%	37.4%	58.9%	45.6%	44.0%	
High school or GED	22.6%	21.2%	17.5%	15.0%	17.7%	
Some college or technical school	29.3%	18.7%	10.6%	12.8%	9.2%	
Bachelor’s degree or higher	21.8%	11.1%	4.8%	5.0%	5.8%	
Unknown	21.4%	11.7%	8.3%	21.7%	23.3%	
*Census division*						<.001
New England	10.2%	4.6%	1.2%	8.9%	12.2%	
Mid Atlantic	17.0%	55.2%	72.0%	24.4%	24.2%	
East North Central	28.5%	6.0%	3.1%	4.7%	6.4%	
West North Central	7.6%	1.2%	0.0%	1.7%	0.6%	
South Atlantic	21.4%	9.4%	13.0%	30.0%	8.7%	
East South Central	1.1%	0.3%	0.0%	0.0%	0.0%	
West South Central	3.2%	4.6%	0.0%	8.6%	2.9%	
Mountain	7.8%	13.6%	0.0%	6.7%	9.1%	
Pacific	3.3%	5.1%	10.8%	15.0%	28.0%	
Unknown	0.1%	0.0%	0.0%	0.0%	7.8%	

*Notes*: *p* values are for comparisons of all five groups. To obtain *p* values, we conducted chi-squared tests for categorical variables and ANOVA for continuous variables. NLW = non-Latino White; DSMP = Diabetes Self-Management Program; PMPD = *Programa de Manoja de la Diabetes*; GED = General Education Diploma.

**Figure 1.**
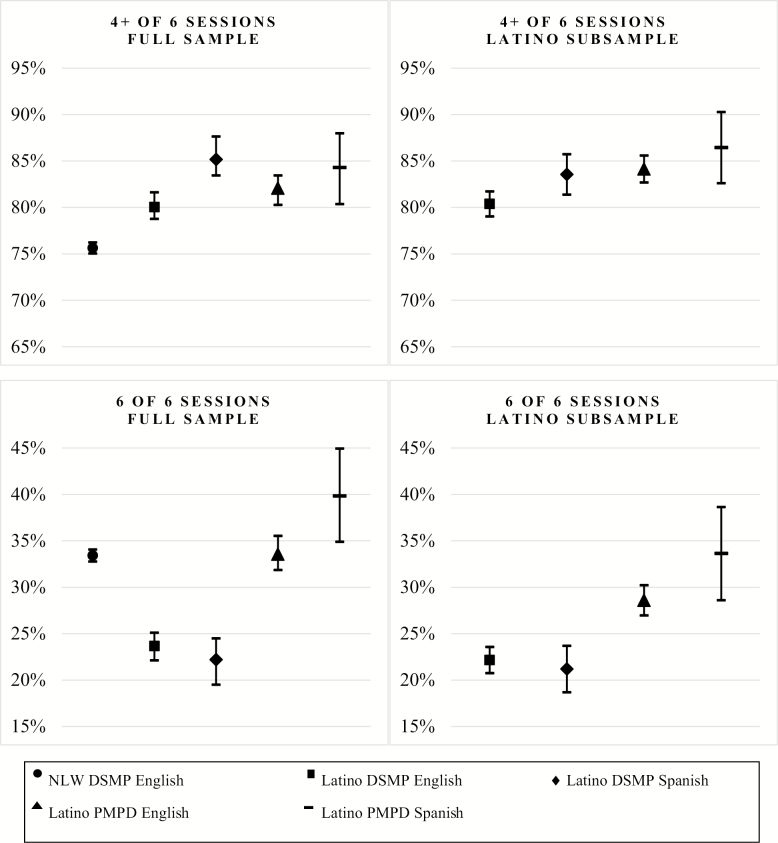
Predicted probabilities of workshop completion and corresponding confidence intervals. *Note*: Whiskers denote 95% confidence intervals estimated from fully adjusted regression models. Predicted probabilities are from fully adjusted regression models.

We estimated linear probability models for the full sample of NLW and Latino participants for each definition of completion (Supplementary Appendix 1 and Figure 1). Predicted probabilities were calculated holding all other variables at their means. We next estimated a model for DSMP English participants only, which included interaction terms for Latino ethnicity and workshop site, because we wanted to determine whether the effects of program delivery site differed for NLW and Latino participants (Supplementary Appendix 2). Based on the joint significance of these interaction terms (*p* < .001), and because we wanted to distinguish the separate effects of program type and language among Latinos, we estimated stratified models for each definition of completion among Latino participants only (Table 2). For all linear probability models, the proportions of predictions that were out of range (<0 or >1) did not exceed 1.66%.

**Table 2.** Factors Affecting the Probability of Completing 4+ of 6 Workshop Sessions, and Completing 6 of 6 Workshop Sessions, Among Latinos

	Model (1)	Model (2)
	4+ of 6 sessions	6 of 6 sessions
*Workshop Participant / Type (reference Latino DSMP English)*		
Latino DSMP Spanish	3.17*	−0.96
	(1.26)	(1.45)
Latino PMPD English	3.77^***^	6.45^***^
	(1.10)	(1.17)
Latino PMPD Spanish	6.07^**^	11.48^***^
	(2.12)	(2.69)
*Workshop Site (reference health care organization)*		
Educational institution	5.71*	6.23
	(2.76)	(3.44)
Faith-based organization	−3.05	1.42
	(2.13)	(2.32)
Residential facility	−10.04^***^	−2.01
	(1.69)	(1.66)
Senior center	−0.56	4.36^***^
	(1.12)	(1.29)
Other site type	−1.65	8.53^***^
	(1.73)	(1.98)
Community center	−4.06^**^	4.46^**^
	(1.53)	(1.70)
Unknown site type	5.24	−6.68
	(12.85)	(13.64)
Age (continuous; 18–110 years)	0.25^***^	0.11^**^
	(0.04)	(0.04)
Female	2.89^**^	0.85
	(0.96)	(1.04)
Number of chronic conditions	−1.19^***^	1.33^***^
	(0.26)	(0.29)
Has health insurance	−5.41*	6.69*
	(2.75)	(2.97)
Lives alone	6.15^***^	−7.78^***^
	(1.12)	(1.26)
*Educational attainment (reference did not graduate high school)*		
High school graduate or GED	−1.36	3.71^**^
	(1.15)	(1.30)
Some college or technical school	−0.20	5.91^***^
	(1.42)	(1.58)
Bachelor’s degree or more	3.95*	6.30^**^
	(1.69)	(1.96)
Unknown educational attainment	−2.53	6.12^***^
	(1.38)	(1.53)
*Census division (reference Pacific)*		
New England	0.96	2.39
	(2.13)	(2.47)
Mid Atlantic	8.01^***^	−13.41^***^
	(1.67)	(1.80)
East North Central	−4.38	0.14
	(2.56)	(2.78)
West North Central	−3.11	−5.93
	(5.26)	(5.55)
South Atlantic	0.19	−1.12
	(1.94)	(2.19)
East South Central	14.32	20.55
	(9.50)	(15.02)
West South Central	−4.28	−8.26^**^
	(3.00)	(3.17)
Mountain	−5.57^**^	−5.66^**^
	(2.07)	(2.18)
Unknown census division	4.28	4.60
	(2.68)	(3.52)
***N***	8,321	8,321

*Notes*: Robust standard errors are in parentheses. *p < .05; **p < .01; ***p < .001.

Coefficients and standard errors are presented as percentage-points.

For Model (1) 1.66% (*n* = 138) of predictions fell outside the 0–1 range.

For Model (2) 0.00% (*n* = 0) of predictions fell outside the 0–1 range.

Other workshop site category includes Area Agency on Aging, county health department, multipurpose social services organization, municipal government, state health department, tribal center, workplace, and other.

DSMP = Diabetes Self-Management Program; PMPD = *Programa de Manejo Personal de la Diabetes*; GED = General Education Diploma.

We computed robust standard errors in all models, using the sandwich estimator. All analyses were performed using STATA version 15 (2017).

## Results

The sample included 23,537 NLW and 8,321 Latino participants (Table 1). Latino participants in all workshop types had larger proportions completing at least four of six sessions compared with NLW participants in DSMP English (75.3% completion); the highest completion proportion was for DSMP Spanish workshop participants (89.7%). The proportion of Latino participants completing 100% (i.e., six of six) of workshop sessions was highest for PMPD English (37.5%), compared to 34.5% for NLW participants in DSMP English and lower proportions for Latino participants in PMPD Spanish, DSMP English, and DSMP Spanish workshops (32.6%, 19.6%, and 16.4%, respectively). On average, NLW participants in DSMP English attended 4.4 sessions. Latino participants attended a similar average number of sessions, ranging from 4.7 in PMPD English to 4.2 in DSMP English. The majority of Latino and NLW participants enrolled in workshops at either health care organizations or senior centers. All χ ^2^ and ANOVA tests indicated significant differences in characteristics among the five groups of NLW and Latino participants.

Figure 1 presents predicted probabilities of workshop completion (both 4+ of 6 sessions and 6 of 6 sessions) and associated confidence intervals, based on multivariate linear probability models (Supplementary Appendix 1). In the full sample, Latino participants had higher probabilities of completing 4+ of 6 sessions for all workshop type/language combinations compared with NLW participants. However, Latino participants in English or Spanish DSMP workshops had a lower probability of completing six of six sessions compared with NLW participants. Latino participants in PMPD Spanish workshops had a higher probability of completing six of six sessions compared with NLW participants.

Among Latino participants only (Figure 1 and Table 2), those enrolled in PMPD English or Spanish workshops had a higher probability of completing 4+ of 6 or 6 of 6 sessions than did those enrolled in DSMP English or Spanish workshops. Completion rates, for 4+ of 6 or all 6 sessions, were highest for PMPD Spanish workshops.

Full regression results among Latino participants only (Table 2) also show that some types of workshop site were associated with completion rates, although the direction of the effect was not always consistent between the two definitions of completion. Latino participants enrolled at educational institutions had a higher probability of completing 4+ of 6 sessions than those enrolled at health care organizations (*p <* .05), while completion rates were lower at residential facilities (*p* < .001) and community centers (*p <* .01). Full completion rates (i.e., six of six sessions) for Latino participants were higher at senior centers (*p <* .001), community centers (*p <* .01), and other site types (*p <* 0.001) than at health care organizations.

Among the Latino subsample (Table 2), older age was significantly associated with higher completion rates using either definition. Women had a higher probability of completing 4+ of 6 sessions. Having more chronic conditions or having health insurance was associated with lower probability of completing 4+ of 6 sessions, but higher rates of completing all 6 sessions. With regard to education level, having a bachelor’s degree was associated with higher rates of completing 4+ of 6 sessions, while participants of all education levels had higher probabilities of completing all six sessions than participants who did not graduate from high school.

## Discussion

This study analyzed data from the CDSME Database, housed at the NCOA, to examine the effect of Latino ethnicity and other factors on completion of the Diabetes Self-Management Program (DSMP) and the *Programa de Manejo Personal de la Diabetes* (PMPD). The analysis pooled a large national sample of DSMP and PMPD participants over a nine-year period. Overall, we found that completion rates differed between Latino and NLW participants, as well as between program types and language of delivery. Some types of workshop site and some participant characteristics were also associated with differences in completion rates, although these patterns were not always consistent for the two definitions of completion that were examined.

Overall, Latino participants in all programs had higher probabilities of completing at least four workshop sessions, and of completing all six sessions in PMPD English, compared with NLW DSMP English participants. However, a stratified analysis showed that Latino participants enrolled in PMPD had a higher probability of completing either at least four or all six sessions than Latino participants enrolled in DSMP. Among Latino PMPD participants, completion rates were higher for Spanish-language workshops, for either definition of completion. Among Latino participants enrolled in DSMP, those enrolled in Spanish-language workshops had a higher probability of completing at least four sessions than those in English-language workshops.

The consistently higher completion rates of PMPD workshops in the stratified analysis suggest that culturally tailored diabetes self-management programs enhance participation by Latino adults. One potential explanation is that these kinds of programs are sensitive to cultural values and beliefs related to diabetes, thus making them more relevant and interesting to Latino participants (see Caballero, 2011).

Stratified analyses also showed that Spanish-language delivery was associated with higher completion rates for PMPD, and to a lesser extent for DSMP. This suggests that linguistic tailoring also enhances Latino adults’ participation in diabetes self-management programs. Linguistic adaptations may use easy-to-understand terminology and wording that make program content accessible to Latino participants with lower educational levels. Linguistically appropriate curricula may improve Latino participants’ health literacy, which has been associated with better health outcomes (Berkman et al., 2004) and motivation and skill for improving self-care (Paasche-Orlow & Wolf, 2007).

The effect of workshop location on Latino participants’ completion rates was less clear than for workshop type and language. Compared to a health care organization (the most common site), the stratified analysis showed workshop delivery at senior centers (the second most common location) was associated with an increased probability that Latino participants completed all six sessions, but did not significantly affect their probability of completing at least four sessions. Interestingly, no workshop site exhibited statistically significant effects of the same sign for both definitions of completion. Further research may be needed to understand whether targeting specific delivery locations could further improve Latino participants’ completion of diabetes self-management programs.

Findings about the effect of some other personal characteristics on workshop completion were puzzling. For both the full sample and the stratified analysis, a larger number of self-reported chronic conditions was associated with lower probability of completing at least four sessions, but higher probability of completing all six sessions. Although health insurance is a longstanding problem in Latino populations (Artiga, Orgera, & Damico, 2019), insurance coverage was high among Latino adults in this study because many were of Medicare age. Nevertheless, in the stratified analysis among Latino participants, having health insurance was associated with a decreased probability of completing at least four sessions, but an increased probability of completing all six sessions. Living alone also exhibited opposite effect signs for the two different definitions of completion. More research may be needed to understand these findings.

Finally, the stratified analysis suggests that better educated Latino participants are more likely to complete diabetes self-management programs. Latino participants with bachelor’s degrees had a higher probability of completing at least four sessions, and graduating high school or having any postsecondary education was associated with higher probability of completing all six sessions. These findings echo recent research showing that not graduating high school was associated with worse attendance and completion of the Chronic Disease Self-Management Program or the DSMP (Bobitt et al., 2019). One question raised by our findings is whether further tailoring diabetes self-management programs for persons with less education may enhance completion rates for Latino participants.

## Limitations

Some limitations of this study should be acknowledged. The data did not include information on participants’ preferred language, which may or may not be congruent with workshop language. Further, DSMP and PMPD were developed in English and Spanish, respectively, for monolingual users of those languages. However, the data show that some participants received DSMP in Spanish or PMPD in English. We do not have further information about these circumstances (i.e., if workshop material was translated before delivery to participants or was delivered by simultaneous translation). Other information on participant characteristics or social context (e.g., depressive symptoms, income, marital status) was limited or had high rates of missingness. However, excluded participants did not appear to differ systematically from included participants with regard to workshop completion or independent variables. Data were not available on Latino participants’ nativity or acculturation level, which has been associated with health-seeking behaviors (see reviews in Abraído-Lanza, Echeverría, & Flórez [2016] and Lara, Gamboa, Kahramanian, Morales, & Hayes Bautista [2005]). We did not restrict our study to participants self-reporting as having diabetes because some participants may have been caregivers or partners of a person with diabetes without having the disease themselves. However, the data did not allow for the identification of dyads to account for potential clustering. Additionally, more than half of participants had missing caregiver status information (i.e., whether they provided care to someone), further restricting our ability to account for this issue in our analyses.

## Implications

Our findings from a multiyear national data set suggest that culturally and linguistically tailored workshop curricula can enhance Latino participants’ completion of diabetes self-management education programs. Further research is needed to understand better the effect of workshop site and other participant characteristics on completion of these programs.

## Supplementary Material

igaa006_suppl_Supplementary_Appendix_1

igaa006_suppl_Supplementary_Appendix_2

## References

[CIT0001] Abraído-Lanza A. F., Echeverría S. E., & Flórez K. R (2016). Latino immigrants, acculturation, and health: Promising new directions in research. Annual Review of Public Health, 37, 219–236. doi: 10.1146/annurev-publhealth-032315-021545 PMC5337110 26735431

[CIT0002] American Diabetes Association (2018). Economic costs of diabetes in the U.S. in 2017. Diabetes Care, 41(5), 917–928. doi: 10.2337/dci18-0007 29567642 PMC5911784

[CIT0003] Artiga S., Orgera K., & Damico A.(February 2019). *Changes in health coverage by race and ethnicity since implementation of the ACA, 2013–2017*. Retrieved from http://files.kff.org/attachment/Issue-Brief-Changes-in-Health-Coverage-by-Race-and-Ethnicity-since-Implementation-of-the-ACA-2013–2017

[CIT0004] Beck J., Greenwood D. A., Blanton L., Bollinger S. T., Butcher M. K., Condon J. E.,… Wang J.; 2017 Standards Revision Task Force. (2017). 2017 national standards for diabetes self-management education and support. Diabetes Care, 40, 1409–1419. doi:10.2337/dci17-0025 28754780

[CIT0005] Berkman N. D., Dewalt D. A., Pignone M. P., Sheridan S. L., Lohr K. N., Lux L.,... Bonito A. J (2004). *Literacy and health outcomes: Summary* Rockville (MD): Agency for Healthcare Research and Quality. Retrieved from https://www.ncbi.nlm.nih.gov/books/NBK11942/

[CIT0006] Bobitt J., Aguayo L., Payne L., Jansen T., & Schwingel A (2019). Geographic and social factors associated with chronic disease self-management program participation: Going the “extra-mile” for disease prevention. Preventing Chronic Disease, 16, E25. doi:10.5888/pcd16.180385 30844360 PMC6429686

[CIT0007] Brown S. A., Garcia A. A., Kouzekanani K., & Hanis C. L (2002). Culturally competent diabetes self-management education for Mexican Americans: The Starr County border health initiative. Diabetes Care, 25, 259–268. doi:10.2337/diacare.25.2.259 11815493 PMC2134805

[CIT0008] Caballero A. E (2011). Understanding the Hispanic/Latino patient. The American Journal of Medicine, 124, S10–S15. doi:10.1016/j.amjmed.2011.07.018 21939793

[CIT0009] Centers for Disease Control (2017, September 18). *Hispanic Diabetes Prevention*. Retrieved June 12, 2019, from Centers for Disease Control and Prevention. Retrieved from https://www.cdc.gov/features/hispanichealth/

[CIT0010] Centers for Disease Control (2018, December 19). *Managing Diabetes | Self-Management Education Programs | Self-Management Education: Learn More. Feel Better*. | CDC. Retrieved June 10, 2019, from https://www.cdc.gov/learnmorefeelbetter/programs/diabetes.htm

[CIT0011] Centers for Disease Control (n.d). *CDC—Ever Attended Diabetes Self-Management Class By Race—Preventive Care Practices— Diabetes DDT*. Retrieved June 10, 2019, from https://www.cdc.gov/diabetes/statistics/preventive/tNewDEduRace.htm

[CIT0012] Erdem E., & Korda H (2014). Self-management program participation by older adults with diabetes: Chronic disease self-management program and diabetes self-management program. Family & Community Health, 37, 134–146. doi:10.1097/FCH.0000000000000025 24569159

[CIT0013] Francis S. L., Noterman A., & Litchfield R. E (2014). Factors influencing Latino participation in community-based diabetes education. Journal of Extension, 52(5), 9.

[CIT0014] Lanting L. C., Joung I. M., Mackenbach J. P., Lamberts S. W., & Bootsma A. H (2005). Ethnic differences in mortality, end-stage complications, and quality of care among diabetic patients: A review. Diabetes Care, 28, 2280–2288. doi:10.2337/diacare.28.9.2280 16123507

[CIT0015] Lara M., Gamboa C., Kahramanian M. I., Morales L. S., & Bautista D. E (2005). Acculturation and Latino health in the United States: A review of the literature and its sociopolitical context. Annual Review of Public Health, 26, 367–397. doi:10.1146/annurev.publhealth.26.021304.144615 PMC5920562 15760294

[CIT0016] Lorig K. R., Ritter P. L., & González V. M (2003). Hispanic chronic disease self-management: A randomized community-based outcome trial. Nursing Research, 52, 361–369. doi:10.1097/00006199-200311000-00003 14639082

[CIT0017] Lorig K., Ritter P. L., Villa F., & Piette J. D (2008). Spanish diabetes self-management with and without automated telephone reinforcement: Two randomized trials. Diabetes Care, 31, 408–414. doi:10.2337/dc07-1313 18096810

[CIT0018] Lorig K., Ritter P. L., Turner R. M., English K., Laurent D. D., & Greenberg J (2016). Benefits of diabetes self-management for health plan members: A 6-month translation study. Journal of Medical Internet Research, 18, e164. doi: 10.2196/jmir.5568 27342265 PMC4950850

[CIT0019] Lorig K., Ritter P. L., Villa F. J., & Armas J (2009). Community-based peer-led diabetes self-management. The Diabetes Educator, 35, 641–651. doi: 10.1177/0145721709335006 19407333

[CIT0020] Mayberry L. S., & Osborn C. Y (2012). Family support, medication adherence, and glycemic control among adults with type 2 diabetes. Diabetes Care, 35, 1239–1245. doi:10.2337/dc11-2103 22538012 PMC3357235

[CIT0021] Ory M. G., Ahn S., Jiang L., Lorig K., Ritter P., Laurent D. D.,… Smith M. L (2013). National study of chronic disease self-management: Six-month outcome findings. Journal of Aging and Health, 25, 1258–1274. doi:10.1177/0898264313502531 24029414

[CIT0022] Osborn C. Y., de Groot M., & Wagner J. A (2013). Racial and ethnic disparities in diabetes complications in the northeastern United States: The role of socioeconomic status. Journal of the National Medical Association, 105, 51–58. doi:10.1016/s0027-9684(15)30085-7 23862296 PMC3852686

[CIT0023] Paasche-Orlow M. K., & Wolf M. S (2007). The causal pathways linking health literacy to health outcomes. American Journal of Health Behavior, 31(Suppl 1), S19–S26. doi:10.5555/ajhb.2007.31.supp.S19 17931132

[CIT0024] Schneiderman N., Llabre M., Cowie C. C., Barnhart J., Carnethon M., Gallo L. C.,… Avilés-Santa M. L (2014). Prevalence of diabetes among Hispanics/Latinos from diverse backgrounds: The Hispanic Community Health Study/Study of Latinos (HCHS/SOL). Diabetes Care, 37, 2233–2239. doi:10.2337/dc13-2939 25061138 PMC4113173

[CIT0025] Self-Management Resource Center (n.d). *Diabetes self-management (DSMP)*. Retrieved from https://www.selfmanagementresource.com/programs/small-group/diabetes-self-management/.

[CIT0026] Smith M. L., Ory M. G., Ahn S., Kulinski K. P., Jiang L., Horel S., & Lorig K (2015). National dissemination of chronic disease self-management education programs: An incremental examination of delivery characteristics. Frontiers in Public Health, 2, 1–7. doi: 10.3389/fpubh.2014.00227 PMC4410345 25964923

[CIT0027] StataCorp (2017). Stata Statistical Software: Release 15. College Station, TX: StataCorp LLC.

[CIT0028] Testerman J., & Chase D (2018). Influences on diabetes self-management education participation in a low-income, Spanish-speaking, Latino Population. Diabetes Spectrum, 31, 47–57. doi: 10.2337/ds16-0046 29456426 PMC5813308

[CIT0029] Turner R. M., Ma Q., Lorig K., Greenberg J., & DeVries A. R (2018). Evaluation of a diabetes self-management program: Claims analysis on comorbid illnesses, health care utilization, and cost. Journal of Medical Internet Research, 20, e207. doi:10.2196/jmir.9225 29934284 PMC6035341

[CIT0030] U.S. Census Bureau. (2018). *Census bureau region and division codes and federal information processing system (FIPS) codes for states* Retrieved from https://www.census.gov/geographies/reference-files/2017/demo/popest/2017-fips.html

[CIT0031] Wen L. K., Parchman M. L., & Shepherd M. D (2004). Family support and diet barriers among older Hispanic adults with type 2 diabetes. Family Medicine, 36, 423–430. 15181555

[CIT0032] Wen L. K., Shepherd M. D., & Parchman M. L (2004). Family support, diet, and exercise among older Mexican Americans with type 2 diabetes. The Diabetes Educator, 30, 980–993. doi:10.1177/014572170403000619 15641619

