# Supplementary material for: Participation of Latinos in the Diabetes Self-Management Program and Programa de Manejo Personal de la Diabetes
Source: Innov Aging. 2020 Mar 18;4(1):igaa006. doi: 10.1093/geroni/igaa006 (PMC7078852; doi:10.1093/geroni/igaa006)
Supplement: igaa006_suppl_Supplementary_Appendix_1 [file igaa006_suppl_supplementary_appendix_1.doc]

Appendix 1. Factors affecting the probability of completing 4+ of 6 workshop sessions, and of completing 6 of 6 sessions, Full Sample

|  | Model (1) | Model (2) |
| --- | --- | --- |
|  | 4+ of 6 sessions | 6 of 6 sessions |
| *Workshop Participant / Type (reference NLW DSMP English* |  |  |
| Latino DSMP English | 4.55^***^ | –9.82^***^ |
|  | (0.84) | (0.88) |
| Latino DSMP Spanish | 9.90^***^ | –11.42^***^ |
|  | (1.15) | (1.36) |
| Latino PMPD English | 6.22^***^ | 0.27 |
|  | (0.91) | (1.05) |
| Latino PMPD Spanish | 8.53^***^ | 6.49^*^ |
|  | (1.98) | (2.60) |
| *Workshop Site (reference healthcare organization)* |  |  |
| Educational institution | 1.06 | 1.70 |
|  | (1.70) | (1.96) |
| Faith-based organization | –1.84 | 1.03 |
|  | (1.13) | (1.23) |
| Residential facility | –7.46^***^ | –1.57 |
|  | (0.88) | (0.92) |
| Senior center | 0.67 | 1.11 |
|  | (0.68) | (0.77) |
| Other site type | –0.59 | 2.30^*^ |
|  | (0.84) | (0.94) |
| Community center | –2.22^**^ | 1.87^*^ |
|  | (0.76) | (0.84) |
| Unknown site type | –3.46 | –1.13 |
|  | (2.28) | (2.40) |
| Age (continuous; 18-110 years) | 0.20^***^ | 0.10^***^ |
|  | (0.02) | (0.02) |
| Female | 1.64^**^ | –0.56 |
|  | (0.54) | (0.59) |
| Number of chronic conditions | –0.37^**^ | 0.44^**^ |
|  | (0.13) | (0.14) |
| Has health insurance | –3.42^***^ | –0.29 |
|  | (0.93) | (1.06) |
| Lives alone | 1.46^**^ | –2.21^***^ |
|  | (0.52) | (0.58) |
| *Educational attainment (reference did not graduate high school)* |  |  |
| High school graduate or GED | –1.67^*^ | 6.22^***^ |
|  | (0.83) | (0.92) |
| Some college or technical school | –0.92 | 6.09^***^ |
|  | (0.86) | (0.94) |
| Bachelor’s degree or more | 1.76 | 5.90^***^ |
|  | (0.91) | (1.01) |
| Unknown educational attainment | –2.13^*^ | 5.26^***^ |
|  | (0.91) | (0.99) |
| *Census division (reference Pacific)* |  |  |
| New England | 1.10 | –2.33 |
|  | (1.28) | (1.44) |
| Mid Atlantic | 5.04^***^ | –8.19^***^ |
|  | (1.14) | (1.25) |
| East North Central | 0.86 | –2.54 |
|  | (1.22) | (1.36) |
| West North Central | 3.23^*^ | –4.50^**^ |
|  | (1.44) | (1.61) |
| South Atlantic | -0.38 | –5.91^***^ |
|  | (1.19) | (1.31) |
| East South Central | 0.80 | –3.31 |
|  | (2.84) | (3.13) |
| West South Central | –1.79 | –4.19^*^ |
|  | (1.71) | (1.86) |
| Mountain | –1.03 | –5.73^***^ |
|  | (1.31) | (1.43) |
| Unknown census division | 4.90^*^ | 6.20 |
|  | (2.41) | (3.26) |
| Lives alone | 1.46^**^ | –2.21^***^ |
|  | (0.52) | (0.58) |
| ***N*** | 31,858 | 31,858 |

Notes: Robust standard errors are in parenthesis. *p < .05; **p < .01; ***p < .001.

Coefficients and standard errors presented as percentage-points.

For Model (1), 0.00% (n=1) of predictions fell outside the 0-1 range.

For Model (2), 0.00% (n=0) of predictions fell outside the 0-1 range.
