# Supplementary material for: Participation of Latinos in the Diabetes Self-Management Program and Programa de Manejo Personal de la Diabetes
Source: Innov Aging. 2020 Mar 18;4(1):igaa006. doi: 10.1093/geroni/igaa006 (PMC7078852; doi:10.1093/geroni/igaa006)
Supplement: igaa006_suppl_Supplementary_Appendix_2 [file igaa006_suppl_supplementary_appendix_2.doc]

Appendix 2. Factors affecting the probability of completing 4+ of 6 workshop sessions and 6 of 6 sessions among DSMP English participants, with interactions for Latino ethnicity and workshop site

|  | Model (1) | Model (2) |
| --- | --- | --- |
|  | 4+ of 6 sessions | 6 of 6 sessions |
| *Ethnicity* |  |  |
| Latino | 9.84^***^ | –21.10^***^ |
|  | (1.25) | (1.30) |
| *Workshop Site (reference healthcare organization)* |  |  |
| Educational institution | 0.53 | –3.13 |
|  | (2.20) | (2.42) |
| Faith-based organization | 0.12 | –0.99 |
|  | (1.34) | (1.47) |
| Residential facility | –5.78^***^ | –3.55^**^ |
|  | (1.05) | (1.11) |
| Senior center | 1.78^*^ | –1.05 |
|  | (0.84) | (0.94) |
| Other site type | 0.78 | –1.03 |
|  | (0.98) | (1.09) |
| Community center | –0.37 | –0.83 |
|  | (0.90) | (1.00) |
| Unknown site type | –2.35 | –2.79 |
|  | (2.33) | (2.46) |
| *Latino & Workshop Site Interactions* |  |  |
| Latino*Educational institution | 0.45 | 32.52^***^ |
|  | (5.98) | (7.85) |
| Latino*Faith-based organization | –8.92^*^ | 21.04^***^ |
|  | (3.89) | (4.29) |
| Latino*Residential facility | –7.89^**^ | 12.93^***^ |
|  | (2.90) | (2.75) |
| Latino*Senior center | –6.63^***^ | 12.81^***^ |
|  | (1.88) | (2.04) |
| Latino*Other site type | –12.56^***^ | 18.49^***^ |
|  | (2.97) | (3.08) |
| Latino*Community center | –10.49^***^ | 17.01^***^ |
|  | (2.36) | (2.51) |
| Latino*Unknown site type | –2.39 | 9.67 |
|  | (13.18) | (13.27) |
| Age (continuous; 18-110 years) | 0.19^***^ | 0.09^***^ |
|  | (0.02) | (0.02) |
| Female | 1.31^*^ | –1.13 |
|  | (0.59) | (0.64) |
| Number of chronic conditions | –0.16 | 0.18 |
|  | (0.14) | (0.15) |
| Has health insurance | –3.22^***^ | –1.76 |
|  | (0.98) | (1.11) |
| Lives alone | 0.51 | –0.64 |
|  | (0.56) | (0.62) |
| *Educational attainment (reference did not graduate high school)* |  |  |
| High school graduate or GED | –2.54^*^ | 4.65^***^ |
|  | (1.06) | (1.12) |
| Some college or technical school | –1.81 | 4.34^***^ |
|  | (1.06) | (1.13) |
| Bachelor’s degree or more | 0.84 | 3.91^***^ |
|  | (1.10) | (1.18) |
| Unknown educational attainment | –2.81^*^ | 2.62^*^ |
|  | (1.15) | (1.21) |
| *Census division (reference Pacific)* |  |  |
| New England | –0.04 | –2.11 |
|  | (1.66) | (1.82) |
| Mid Atlantic | 3.67^*^ | –3.77^*^ |
|  | (1.53) | (1.69) |
| East North Central | 0.56 | –0.91 |
|  | (1.56) | (1.73) |
| West North Central | 2.79 | –2.38 |
|  | (1.74) | (1.94) |
| South Atlantic | –1.39 | –4.30^*^ |
|  | (1.56) | (1.70) |
| East South Central | 0.31 | –1.04 |
|  | (3.00) | (3.29) |
| West South Central | –1.20 | –0.49 |
|  | (2.03) | (2.22) |
| Mountain | –1.13 | –3.85^*^ |
|  | (1.68) | (1.82) |
| Unknown census division | 14.87 | 3.73 |
|  | (8.10) | (12.04) |
| ***N*** | 27,199 | 27,199 |

Notes: Robust standard errors are in parenthesis. *p < .05; **p < .01; ***p < .001.

Coefficients and standard errors presented as percentage-points.

For Model (1), 0.00% (n=0) of predictions fell outside the 0-1 range.

For Model (2), 0.00% (n=0) of predictions fell outside the 0-1 range.
